# DeepM6ASeq: prediction and characterization of m6A-containing sequences using deep learning

**DOI:** 10.1186/s12859-018-2516-4

**Published:** 2018-12-31

**Authors:** Yiqian Zhang, Michiaki Hamada

**Affiliations:** 1 Department of Electrical Engineering and Bioscience, Faculty of Science and Engineering, Waseda University, 55N-06-10, 3-4-1 Okubo Shinjuku-ku, Tokyo, 169-8555 Japan; 2 AIST-Waseda University Computational Bio Big-Data Open Innovation Laboratory (CBBD-OIL), 3-4-1, Okubo Shinjuku-ku, Tokyo, 169-8555 Japan; 3 Artificial Intelligence Research Center, National Institute of Advanced Industrial Science and Technology (AIST), 2-41-6 Aomi, Koto-ku, Tokyo, 135-0064 Japan; 4 Institute for Medical-oriented Structural Biology, Waseda University, 2-2, Wakamatsu-cho Shinjuku-ku, Tokyo, 162-8480 Japan; 5 Graduate School of Medicine, Nippon Medical School, 1-1-5, Sendagi, Bunkyo-ku, Tokyo, 113-8602 Japan

**Keywords:** RNA modification, N6-methyladenosine, Deep learning

## Abstract

**Background:**

N6-methyladensine (m6A) is a common and abundant RNA methylation modification found in various species. As a type of post-transcriptional methylation, m6A plays an important role in diverse RNA activities such as alternative splicing, an interplay with microRNAs and translation efficiency. Although existing tools can predict m6A at single-base resolution, it is still challenging to extract the biological information surrounding m6A sites.

**Results:**

We implemented a deep learning framework, named DeepM6ASeq, to predict m6A-containing sequences and characterize surrounding biological features based on miCLIP-Seq data, which detects m6A sites at single-base resolution. DeepM6ASeq showed better performance as compared to other machine learning classifiers. Moreover, an independent test on m6A-Seq data, which identifies m6A-containing genomic regions, revealed that our model is competitive in predicting m6A-containing sequences. The learned motifs from DeepM6ASeq correspond to known m6A readers. Notably, DeepM6ASeq also identifies a newly recognized m6A reader: FMR1. Besides, we found that a saliency map in the deep learning model could be utilized to visualize locations of m6A sites.

**Conculsion:**

We developed a deep-learning-based framework to predict and characterize m6A-containing sequences and hope to help investigators to gain more insights for m6A research. The source code is available at https://github.com/rreybeyb/DeepM6ASeq.

**Electronic supplementary material:**

The online version of this article (10.1186/s12859-018-2516-4) contains supplementary material, which is available to authorized users.

## Background

More than 100 types of RNA modification have been discovered in eukaryotic RNAs [1]; among them, N6-methyladenosine (m6A) is a common and abundant RNA modification type found in various species, such as human, mouse and yeast [2–4]. m6A is preferentially located near 3’ untranslated regions (3’ UTR) and its nearby sequences mostly conform to certain motifs, i.e., DRACH (where D = A, G or U; R = A or G; H = A, C or U) in the mammalian genome [5] and RAC in the yeast genome [6]. m6A is involved in diverse RNA activities including alternative splicing [7], an interplay with microRNAs [8] and translation efficiency [9]. In addition, m6A has been linked with caner progression. It is reported that METTL3 and METTL4, which are both m6A-forming enzymes, have an impact on differentiation and apoptosis of human myeloid leukemia cell lines [10, 11].

m6A can be detected in a high-throughput manner owing to the rapid development of high-throughput sequencing technologies. m6A-Seq and Methylated RNA immunoprecipitation sequencing (MeRIP-Seq) [2, 3] are the main sequencing methods for detection of genomic regions with m6A sites via antibody capturing. Recently, m6A individual-nucleotide-resolution cross-linking and immunoprecipitation (miCLIP-Seq) enables detection of m6A at single-base resolution [5, 12]. Several bioinformatics tools have been developed to predict m6A sites in different species, e.g., m6Apred [13] and iRNA-Methyl [14] for the yeast genome, SRAMP [15] for the mammalian genome. These tools mainly apply existing knowledge as feature input such as a combination of k-mers and chemical properties to build models using random forest (RF) or support vector machine (SVM) algorithm. Although these tools can predict single-base m6A, the biological information surrounding m6As is still limited; this situation poses a challenge for researchers. Therefore, here we implemented a deep-learning-based framework, named DeepM6ASeq, to predict m6A-containing sequences and characterize biological features surrounding m6A. In recent years, deep learning became an state-of-the-art technology and is now employed more and more in the field of biology [16–18]. The strength of deep learning is not only in its better prediction power (in comparison with traditional machine learning classifiers), but also its ability to recognize motifs in genomic sequences. Because miCLIP-Seq data revealed precise locations of m6A sites, we explored on such data by utilizing convolutional neural network (CNN) layer as a motif detector to characterize biological features surrounding m6A, then capturing m6A’s positional preference out of the deep learning model we built. In addition, we made use of a saliency map to visualize locations of m6A sites in the sequences. The development of DeepM6ASeq, model performance and analysis of biological information will be discussed in details in the following sections.

## Methods

### Datasets

#### The miCLIP-Seq dataset

Given that miCLIP-Seq data can pinpoint m6A sites at single-base resolution, these data provide us with ideal conditions to study sequences surrounding m6A sites. We collected miCLIP-Seq data from human, mouse and zebrafish [5, 12, 19]. Human and mouse data are from the same source as SRAMP, which included five cell line and tissue types, that is A549, CD8T, HEK293, brain and liver. For zebrafish, the data consisted of two biological replicates from embryonic stem cells.

For positive samples, we defined sequences with the window size of 101 bp containing m6A sites. First, all m6A sites were mapped to the longest transcripts of genes using the ENSEMBL database (release 91, http://www.ensembl.org/). Then, we randomly located m6A sites in the fixed-size windows and extracted the surrounding sequences with length up to 101 bp (if m6A sites are near a terminus of a transcript, we sliced 101-bp-size windows from the terminus). To avoid sample redundancy (because m6A sites are reported to cluster together [2]), before randomly locating we merged m6A sites within 50 bp and chose the centered one among the merged sites. Because zebrafish data consisted of two replicates, we chose common sites as positive samples.

For negative samples, we used nearby windows (with the same fixed window size) not containing any m6A sites. The nearby negative controls are from the windows 100 bp upstream or downstream the positive windows; these windows are generated by a stride of 10 bp and 100 steps. We chose the closest one for each positive sample. (If there were two closest ones on both sides of a positive sample, we randomly picked one of the two.) In rare cases, there were no control windows nearby because m6A sites are mapped to very short transcripts. Nevertheless, the ratio of positive to negative samples was approximately 1:1. For each species, we split the dataset into an 80% part (as training data) and a 20% part (as independent test data). The dataset information is listed in Table 1.

**Table 1:** A summary of dataset size

	Training	Independent test
Human	49050	12611
Mouse	37716	9401
Zebrafish	22108	5651

#### The m6A-Seq dataset

To test our model on real peaks data, we used m6A-Seq data from the HepG2 cell line and human brain (two different cell types from those used in the model) from Dominissini’s study [3] and processed this dataset according to their protocol [20]. For positive samples, we retrieved the top 1000 positive peaks detected by MACS [21] with the highest fold enrichment and the false discovery rate (FDR) ≤ 0.05. We extracted sequences of 101 bp around the peak summits and overlapped these regions with peaks from MeT-DB database [22] (The MeT-DB peak score greater than 6 was required, which is the median score for human data.) to obtain reliable m6A-containing sequences. As negative samples, we used negative peaks detected by MACS (MACS identifies negative peaks by swapping immunoprecipitation samples and control samples) and split each peak into bins with a size of 101 bp(because HepG2 has limited negative peaks, we used a sliding window with a step of 20 bp when spliting peaks for data augmentation). We chose bins overlapping with exon regions and not overlapping with peaks from MeT-DB database. To evaluate the generalization of our model and to conduct a fair comparison with SRAMP, we used CD-HIT [23] to remove test sequence redundancy with the training data of both our model and SRAMP at an 80% similarity threshold, which is the lowest threshold provided by CD-HIT. Besides, we kept only sequences with DRACH motifs because SRAMP scans only A sites with DRACH motifs in given sequences. Finally, we got 663 positive samples and 413 negative samples in total.

### Models

#### The development of deep learning models

**Fig. 1:**
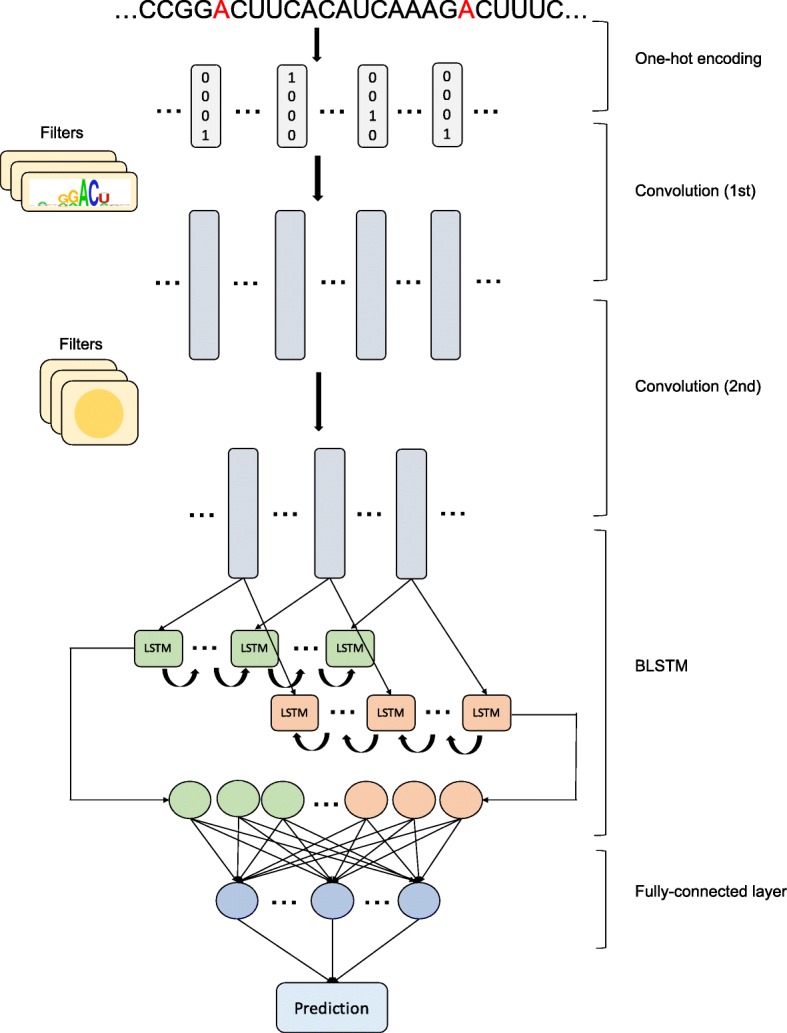
A graphic illustration of DeepM6ASeq model structure. The genome sequence (A in red represents an m6A site) is first one-hot encoded as input, then the input is sequentially fed into two layers of CNN in order. The first CNN layer functions as a motif detector while the second CNN layer captures features of a higher level. After the CNN layers is one BLSTM layer to capture sequential order. The output units of the BLSTM layer are followed by the fully connected layer, and finally the model outputs the prediction result

The sequences were one-hot encoded as inputs with the padding of half filter size on each side, that is, A, C, G, U, and N were encoded as (1,0,0,0), (0,1,0,0), (0,0,1,0), (0,0,0,1), and (0,0,0,0) respectively. The main structure of our deep learning model consists of two layers of CNN [24], one bidirectional long short-term memory (BLSTM) layer [25] and one fully connected (FC) layer as presented in Fig. 1. The first convolution layer works as a motif detector, while the second convolution layer captures higher-level features. The BLSTM layer is useful to get sequential-order information embedded in the sequences.

During the process of model construction, we chose the filter sizes of 10 and 5, the filter numbers of 256 and 128 for each convolution layer. The activation function for CNN layers is rectified linear unit (ReLU), tanh for the BLSTM layer and sigmoid activation after the FC layer to obtain prediction output. Additionally, we applied batch normalization and dropout [26] after each convolutional procedure to accelerate training and avoid overfitting separately. We used binary cross entropy as a loss function to measure the difference between the target and the predicted output and Adam as an optimization algorithm. The deep learning framework is implemented using Pytorch (https://pytorch.org).

There are three phases during the process of model building. First, we performed five-fold cross-validation on training data for optimization of hyperparameters. In this phase, we used the grid-search strategy for optimization of hyperparameters. The details of tuning parameters are given in Additional file 1: Table S1. Then, we used 1/8 of training data, which equals to 10% of the whole dataset, as validation data and fed the best parameters from the previous phase to the training phase. In the last phase, we applied our model to the independent dataset. We selected a batch size of 256, 50 maximum epochs and an early stopping strategy of patience to 5 in the first two phases.

#### Conversion of filters to motifs

We employed the method from previous papers [16, 18] to convert filters to motifs in position weight matrix (PWM) format. For each input sequence, the subsequence with the filter length that responds to the corresponding filter maximally is extracted in a one-hot encoded matrix, which is then multiplied by the responding score from ReLU in the first CNN layer as follows

1 $$\begin{array}{*{20}l} M_{l,4}^{(k)}=\sum\limits_{i=1}^{n}\alpha_{i}^{(k)}X_{l,4}^{(i)} \end{array} $$

where *X* is the subsequence matrix, *α* is the responding score, *l* represents the filter length, *k* denotes the motif detector, and *n* is the number of input sequences. The cumulative matrix of these subsequences forms a PWM, each element of which is then normalized as described below

2 $$ m_{p,q}=\frac{m_{p,q}}{{\sum\nolimits}_{q=1}^{4}m_{p,q}}   $$

where *m* stands for each element in *M*, and *p* and *q* are the row number and column number respectively.

#### The saliency map

A saliency map is used to determine which nucleotide makes the most contribution to the prediction score for a class (*S*_*c*_). We calculated the saliency map according to the method described by Lanchantin et al. [27]. First, the class score could be approximated with a liner function by computing the first-order Taylor expansion:

3 $$\begin{array}{*{20}l} S_{c}(X)\approx w(X)^{T}X+b. \end{array} $$

Then, for a given sequence *X* in one-hot encoding, the saliency score *S* was obtained by a point-wise multiplication of the absolute value of a derivative of *S*_*c*_(*X*) and its one-hot encoding formally expressed as

4 $$\begin{array}{*{20}l}  w(X)=\frac{\partial S_{c}}{\partial X} \end{array} $$

and

5 $$\begin{array}{*{20}l}  S(X)=\left | w(X) \right |*X \end{array} $$

## Results

***Derivation of other classifiers***

We built models of RF, Logistic Regression (LR) and SVM on mammalian dataset using sklearn (http://scikit-learn.org). For RF and LR, the feature inputs were normalized counts of kmers of 1-5. For SVM, the feature inputs were commonly used 4-kmer for saving training time. We applied the grid-search strategy on hyperparameter optimization for each classifier and chose the parameters with the best performance. The parameters used in the grid-search were listed in Additional file 1: Table S2.

***Evaluation metrics***

To measure performance of the models, we calculated accuracy, sensitivity, specificity, the F1-score and the Matthews correlation coefficient (MCC) as follows:

$$ \begin{aligned} Accuracy = \frac{TP + TN}{TP + TN + FP + FN}\\ Sensitivity = \frac{TP}{TP+FN}\\ Specificity = \frac{TN}{TN+FP}\\ F1\mathrm{-}score = \frac{2TP}{2TP+FP+FN}\\ \end{aligned}  $$

6 $$ {\begin{aligned} MCC = \frac{TP\times TN-FP\times FN}{\sqrt{(TP+FP)\times(TP+FN)\times(TN+FP)\times(TN+FN)}} \end{aligned}}  $$

where TP is true positive, TN is true negative, FP is false positive and FN is false negative. Additionally, we plotted Receiver Operating Characteristic (ROC) curves and Precision-Recall (PR) curves and calculated the areas under the curves, which are denoted by AUROC and AUPR, respectively.

### Prediction of m6A-containing sequences

#### Model training and hyperparameter optimization

We used the mammalian dataset that consists of both human and mouse miCLIP-seq data, for optimizing the hyperparameters during the development of the model. The details of the model development are described in the Materials and Methods section. In brief, we built a deep-learning-based model that mainly consists of two CNN layers, one BLSTM layer and one FC layer, to predict whether a sequence contains m6A sites. During hyperparameter optimization, the grid-search strategy was applied to find the best parameter combination of maxpooling size, dropout rate, learning rate, units of the BLSTM layer and the FC layer. The metrics of mean performance for different parameters settings are shown in Additional file 1: Table S3. We found that no maxpooling, a higher dropout rate and a more complicated model structure contribute to the improvement of performance. Then, we chose the best parameter setting to train the model on the mammalian validation dataset and got AUROC = 0.843 and AUPR = 0.832 for validation as illustrated in Additional file 2: Figure S1.

#### The comparison of DeepM6ASeq with other classifiers

**Fig. 2:**
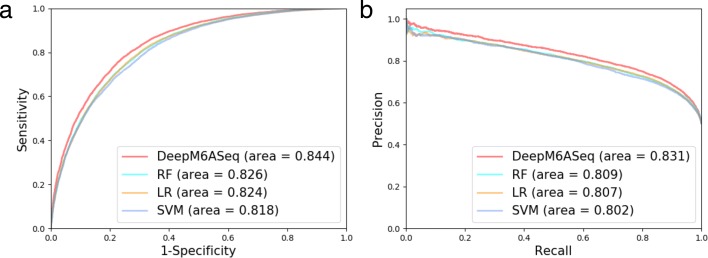
The comparison of DeepM6ASeq with other classifiers, including random forrest (RF), logistic regression (LR) and support vector machine (SVM), on the mammalian independent dataset. The performance is presented as (**a**) a plot of ROC and (**b**) a graph of precision-recall curves

We evaluated our mammalian model on the mammalian independent dataset and compared the model with other classifiers, including LR, RF and SVM. The hyperparameter optimization was performed too for each of these traditional classifiers which as presented in Additional file 1: Table S2. DeepM6ASeq showed improved performance, with AUROC = 0.844 and AUPR = 0.831 (Fig. 2). The performance metrics are listed in the Table 2 in which DeepM6ASeq ranks first in terms of all the evaluation metrics.

**Table 2:** Performance metrics for comparison of DeepM6ASeq with other classifiers on the mammalian independent dataset

	Accuracy	F1-score	AUROC	AUPR	MCC
DeepM6ASeq	**0.764**	**0.762**	**0.844**	**0.831**	**0.528**
Random forest	0.747	0.756	0.826	0.809	0.494
Logistic regression	0.743	0.736	0.824	0.807	0.487
Support vector machine	0.736	0.732	0.818	0.802	0.472

The highest value for each accuracy measure is highlighted in bold

To check the statistical significance of the improved performance, we applied the t-test on ROC values from five-fold cross-validation results between DeepM6ASeq and other three classifiers. The mean and standard deviation of ROC values were 0.8504±0.0025, 0.8304±0.0030, 0.8298±0.0031, 0.8258±0.0037 for DeepM6ASeq, RF, LR and SVM respectively. All the t-test yielded p-value less than 4.5*10e-6, which is indicative of DeepM6ASeq’s superiority. Besides, we also tested the mammalian model on an unbalanced mammalian dataset, consisting of the closest nearby windows without any m6A sites on both sides of the positive samples; this arrangement results in the ratio of positives to negatives nearly 1:2. The performance metrics of the mammalian model on the unbalanced independent dataset are compiled in the Additional file 1: Table S4: DeepM6ASeq showed the stable performance on the unbalanced dataset and still outperformed the other classifiers. Our deep learning model has its strengths: it does not require existing knowledge as input and extracts the features automatically, whereas traditional classifiers need predefined features. Additionally, DeepM6ASeq also takes into account the sequential-order information by applying the BLSTM layer. In summary, our results indicate that DeepM6ASeq performs better than the other three algorithms with only sequence-based feature input.

#### DeepM6ASeq performance on m6A-Seq data

**Fig. 3:**
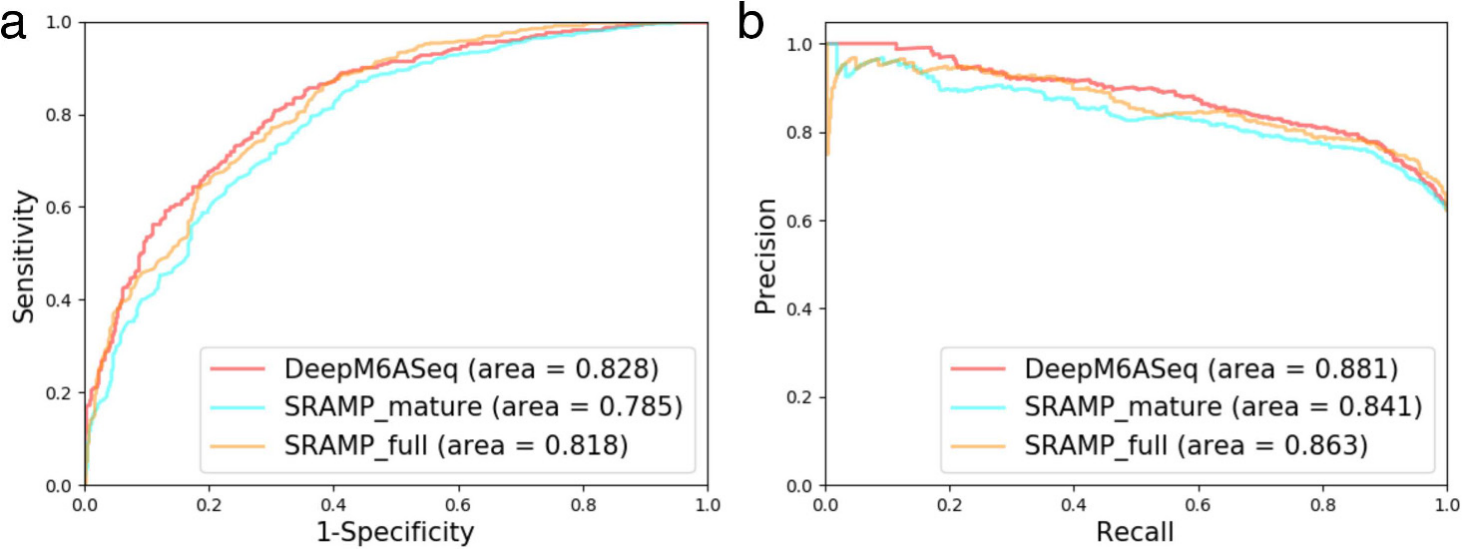
Comparison of DeepM6ASeq with SRAMP in full mode and mature mode (the full mode for whole-transcript sequences and the mature model for cDNA sequences) on the m6A-Seq dataset. The performance is shown as **a**) a plot of ROC and **b**) a graph of precision-recall curves

Given that our independent test samples are generated by a stochastic process, we wondered how our model performs on the real m6A-Seq peak data. We retrieved m6A-Seq peak data from HepG2 cell line and human brain (see the Materials and Methods section) and compared the performance of the mammalian model with that of SRAMP, which is also a sequence-based predictor built for the mammalian genome. Both the full mode and mature mode of SRAMP were compared, where the full mode is for whole transcripts and the mature mode for cDNA sequences. We used SRAMP’s highest score among all the scores for predicted A sites as the prediction score for a given sequence. DeepM6ASeq showed better performance in terms of AUROC and AUPR as presented in Fig. 3, and we list performance metrics in Table 3. Our results indicate that DeepM6ASeq is competitive in predicting m6A-containing sequences.

**Table 3:** Performance metrics for comparison of DeepM6ASeq with SRAMP on the m6A-Seq dataset

	Accuracy	F1-score	AUROC	AUPR	MCC
DeepM6ASeq	**0.763**	0.808	**0.828**	**0.881**	**0.499**
SRAMP-Mature	0.732	0.787	0.785	0.841	0.428
SRAMP-Full	0.762	**0.824**	0.818	0.863	0.483

The highest value for each accuracy measure is highlighted in bold

#### Cross-species performance

We built models for human, mouse and zebrafish separately. The cross-species performance is illustrated in Additional file 2: Figure S2. As expected, the cross-species prediction was stable between human and mouse; howerver, there was a gap in the prediction of the mouse and human dataset by the zebrafish model and vice versa. Because the zebrafish dataset is from one cell line, it is possible that models from other species have limitations in terms of generalization due to the cell-line specificity.

### Biological information on sequences surrounding m6A sites

#### Learned motifs for each species

**Fig. 4:**
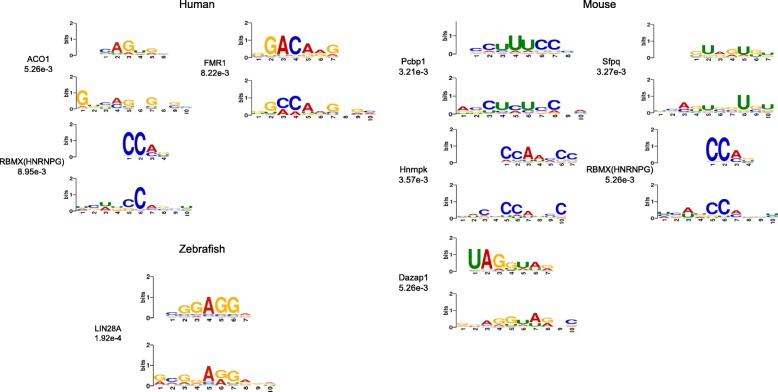
Significant learned motifs (E-value < 0.01) in human, mouse and zebrafish. The learned motifs from the first CNN layer of each species model are aligned with known motifs by means of TOMTOM. For each aligned result, the upper panel is the known motif, while the bottom panel is the learned motif. The names of known motifs and the significant scores (E-value) are shown on the side

The first CNN layer of the deep learning model is a motif detector, thus we wondered what biological information could be captured by models for different species. The filters of the first CNN layer are converted to the motifs in the ways described in refs. [16, 18], in which we extracted the subsequences with the filter length that respond to the filters maximally from positive training sequences and converted these subsequences to PWMs.These learned motifs were aligned to known motifs using TOMTOM [28]. Under the threshold of E-value =0.05, were 18, 21, 15 out of 256 convolutional filters (7%, 8% and 6%) corresponding to known motifs for human, mouse and zebrafish respectively. As depicted in Fig. 4, among the most significant motifs (E-value ≤ 0.01), we found Rbmx (also know as HNRNPG) in both the human and mouse model, which is a known m6A reader [29]. Interestingly, the human predictor detects FMR1, which is a recently discovered m6A reader [30]. FMR1 has been detected in the mouse predictor, albeit not so significant as that in the human predictor (E-value = 0.013). In the zebrafish predictor, the most significant motif was LIN28A, which is one of the core pluripotency regulators. Because the zebrafish data came from embryotic cell line, this outcome is consistent with m6A’s role in controlling cell fate development [31]. The results above suggest that DeepM6ASeq could capture meaningful biological information surrounding m6A sites which is also consistent with biological experiments. Furthermore, we used RSAT [32] for clustering motifs and got 161, 158 and 177 clusters separately for human, mouse and zebrafish(Additional file 2: Figure S3). The detailed information on motifs and clusters information can be found at https://github.com/rreybeyb/DeepM6ASeq.

#### Location preference for m6A-containing sequences

m6A is characterized by enrichment near 3’ UTR of transcripts, thus we wanted to know if our predictor could capture such location information. We performed the position analysis in a way without prior knowledge in which we split the transcripts of the independent test dataset into bins of 101-bp size, get bins with confident prediction scores and check if these bins have location preference with regard to the transcript structure. We established three confidence categories (moderate, high and very high) for prediction scores, which corresponds to 90%, 95% and 99% specificity respectively in the validation datasets (see Additional file 1: Table S5).

First, we computed the percentage of potential m6A-containing bins with scores above moderate confidence in the bins of the the whole transcripts, all exons and last exons. The result indicated that these potential m6A-containing bins are not enriched in the last exons. This finding suggests that sequences with a potential to contain m6A sites are widely distributed along the exons of transcripts (Additional file 2: Figure S4).

**Fig. 5:**
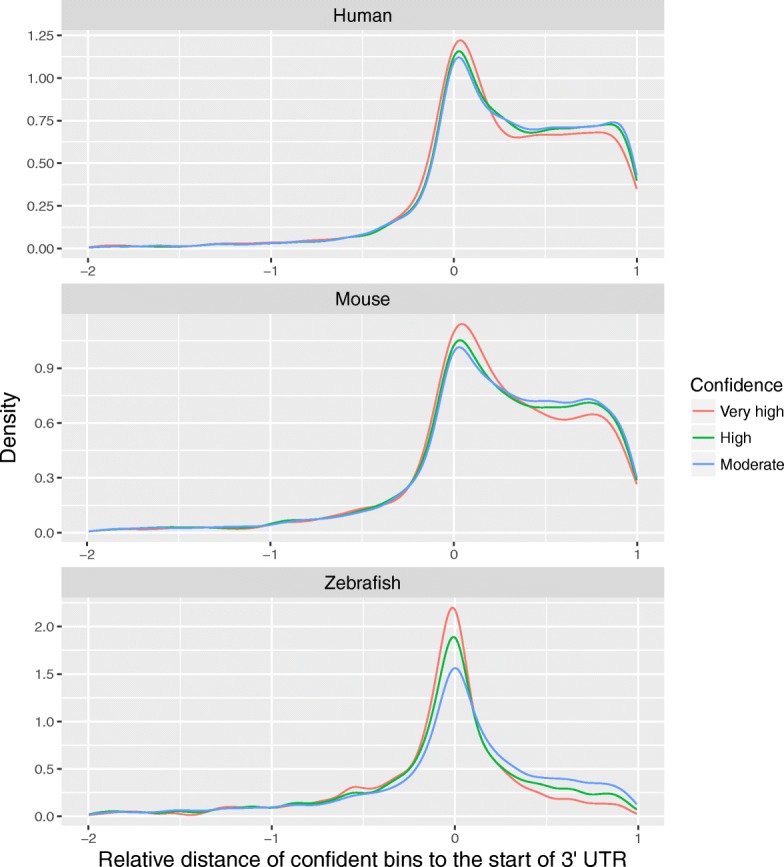
Position profiles of potential m6A-containing bins with a size of 101bp in the last exons for human, mouse and zebrafish. The X-axis represents the relative distances from m6A-containing bins in the last exons to 3’ UTR, which is the distance from bins’ center to the start of 3’ UTR normalized to the length of the 3’ UTR. Different colors of lines represent confidence levels from moderate to very high, which corresponds to 90%, 95% and 99% specificity respectively

Then, we checked the relative position of bins of moderate-to-very high confidence in the last exons toward 3’ UTR. We profiled the relative distances from the center of these bins to the start of 3’ UTR as shown in Fig. 5. (The distance was normalized to the length of 3’ UTR.) The relative distance less than -2 is not shown in the figure because some values are huge owing to the small size of 3’ UTR, and because such bins account for less than 3% in the mammal and 7% in the zebrafish. This finding suggests that our predicted potential m6A-containing bins were enriched near the start of 3’ UTR as the confidence level increased. This result is consistent with the known m6A location bias.

In summary, our location analysis indicates that sequences with a potential to contain m6A sites are widely distributed along the exons of transcripts, in particular, the potential m6A-containing sequences in the last exons are preferentially located near the start of 3’ UTR.

#### The saliency map for visualizing m6A sites

A saliency map is commonly used in computation version for showing each pixels’ unique quality. In the context of a genome sequence, a saliency map can measure the nucleotide importance which can have an impact on the prediction scores. Given that we had precise m6A locations from miCLIP-Seq data, we were curious whether locations of m6A sites could be uncovered by way of a saliency map. We obtained saliency maps for potential m6A-containing sequences in the independent datatsets with prediction scores with higher-than-moderate confidence via the method described by Lanchantin et al. [27], which, in briefly, performs point-wise multiplication of the absolute derivative of the input sequences from back-propagation and their one-hot encoding.

First, we checked the distribution of the types of the most salient nucleotides in the sequences. We extracted the nucleotides with the highest saliency score for each sequence and plotted the distribution. As shown in Additional file 2: Figure S5, nucleotide type A accounted for the majority among all the most salient nucleotides. For those most salient nucleotides rather than A, we plotted the distribution of the distance from these non-A nucleotides to the closest mapped miCLIP m6A sites as depicted in Additional file 2: Figure S6, in which the majority of these most salient non-A nucleotides are located near mapped miCLIP m6A sites.

After that, we wondered how many of these most salient A*s* are overlapped with known m6A sites. Our result revealed that nearly 40–50% of these A*s* belong to known m6A sites from miCLIP-data (Additional file 2: Figure S7). Besides, some of non-miCLIP m6A could be mapped to the predicted m6A sites in the Met-DB single-base m6A database. Although in zebrafish, the most salient A*s* overlapping neither with miCLIP-Seq data nor Met-DB are more than those in human and mouse, actually, over 30% of these A*s* belongs to the miCLIP m6A sites of one of the replicate zebrafish samples.

**Fig. 6:**
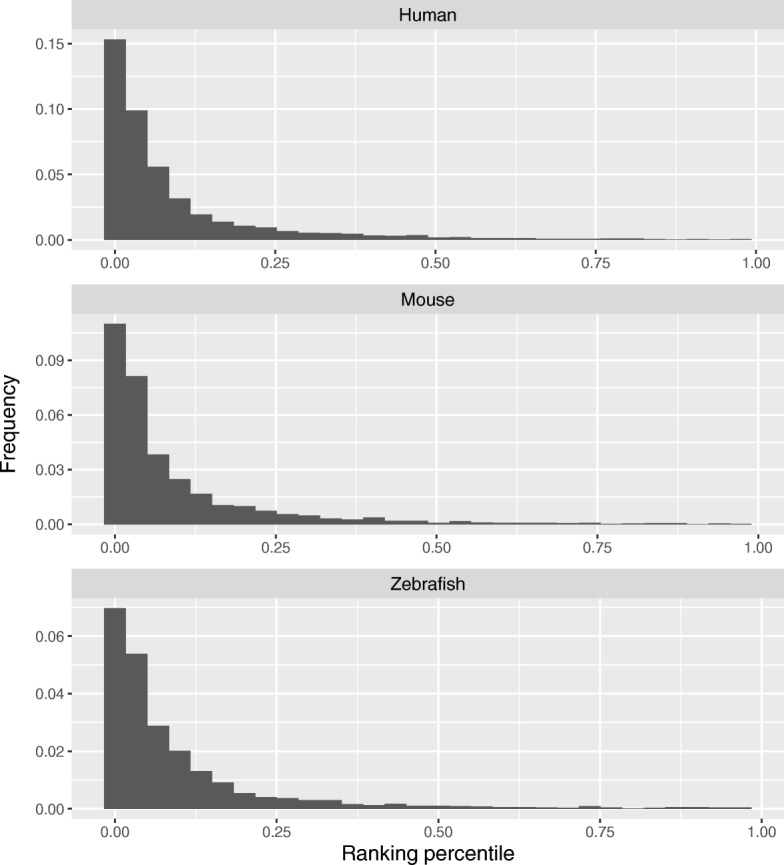
The distribution of ranking percentiles of saliency scores of miCLIP-Seq m6A sites in human, mouse and zebrafish. The X-axis is the ranking percentile of saliency scores of miCLIP-Seq m6As among those of all the A*s* in the independent test sequences with confidence above a moderate threshold

**Fig. 7:**
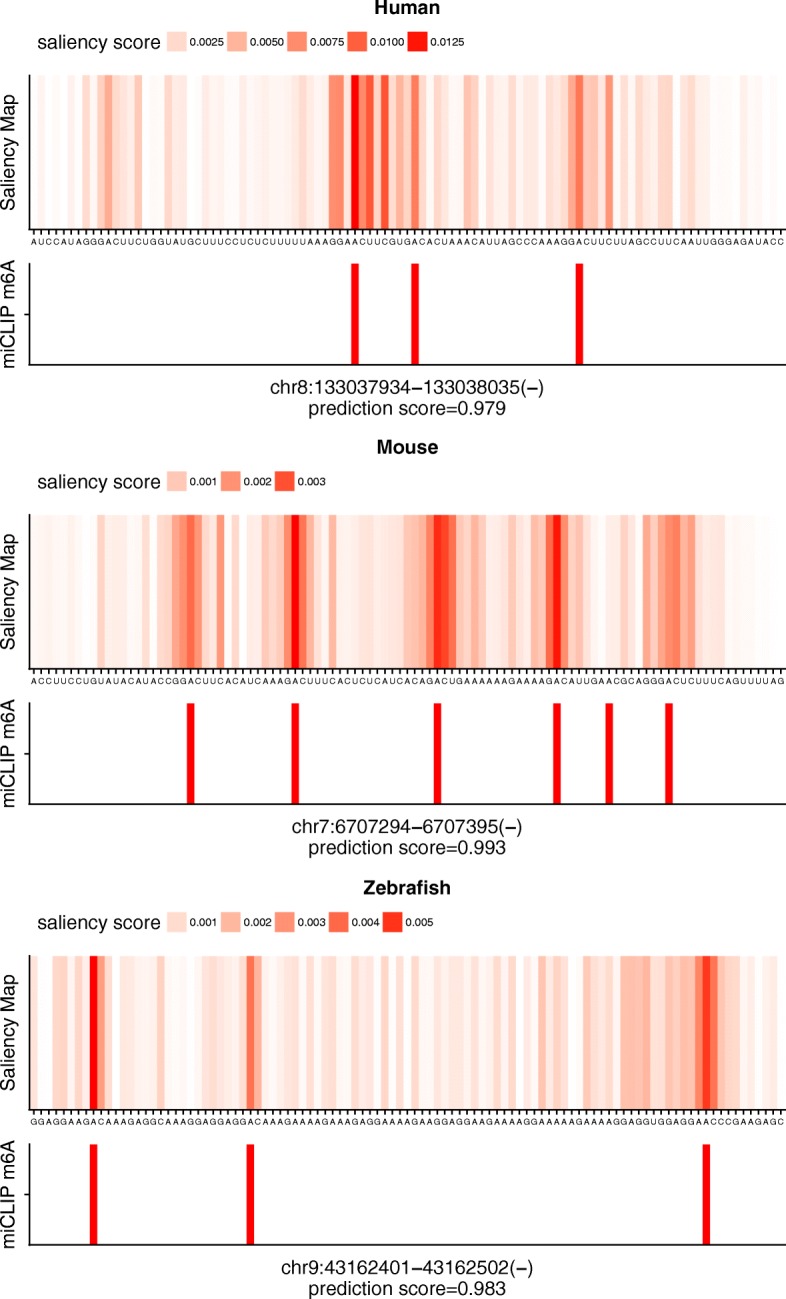
Examples of saliency maps in human, mouse and zebrafish. For each species, the upper panel presents saliency scores of each nucleotide in the sequence and the bottom panel reveals the locations of mapped miCLIP-m6A sites. The position information and the prediction scores for the sequences are listed at the bottom

Even though most salient nucleotides are overlapped with known miCLIP m6A sites to some extent, we wonder if these known miCLIP m6A sites have higher saliency scores as compared to the other A*s* in the sequences. Thus, we evaluated the ranking percentile of the saliency scores for known miCLIP-Seq m6A sites in the sequences. We found that most of miCLIP m6A sites ranked ahead as shown in Fig. 6. We also provide examples of visualization of saliency maps as illustrated in Fig. 7, in which obvious red bands for A*s* are consistent with mapped miCLIP-Seq m6A sites. In the saliency map example for mouse, even though one miCLIP-Seq m6A was missing, we found that this m6A site conforms to a non-DRACH motif and is located between two more significant m6A sites. All the above results indicate that a saliency map could serve as an efficient tool to visualize locations of m6A sites.

## Discussion and conclusion

We propose DeepM6ASeq as a framework useful for identifying m6A-containing sequences. Nonetheless, we have some thoughts about the future research. First, although the zebrafish model has higher predictive power, biological information extracted from this model is limited probably due to the single source of the cell type. We expect additional miCLIP-Seq data to become available for zebrafish in the future to improve the current model and provide more biological information. Second, because the second CNN layer detects the combination of motifs at a higher level, it would be interesting to explore what the deep learning model could detect in this layer. An alternative approach is to apply word-embedding, a strategy widely used in the natural language processing. In this way, input sequences can be converted to words and then a deep learning model can be built to discern some patterns among the sequence words. The word-embedding strategy has been utilized for identifying chromatin accessibility [33]. Finally, to characterize biological features surrounding m6A sites in some way without prior knowledge, we employed all the m6A sites rather than limiting ourselves to m6A sites with DRACH motifs. We believe that deep leaning method may also exert its power for predicting single-base m6A sites with DRACH motifs, in particular combined with other features such as secondary structure and conservation score.

In conclusion, we developed DeepM6ASeq, a model based on deep learning framework, to predict m6A-containing sequences and characterize biological features surrounding m6A sites. DeepM6ASeq showed better performance as compared to other machine learning classifiers and is competitive at predicting m6A-containing sequences. In addition, DeepM6ASeq can recognize the position preference of sequences harboring m6A sites. All these data corroborate the effectiveness of our models. Furthermore, taking advantage of function of motif detectors and saliency maps in the deep learning model, DeepM6ASeq learned a newly recognized m6A reader, FMR1 and helped to visualize mapped and potential m6A sites. We hope that DeepM6ASeq will provide more insights for m6A research.

## Additional files

Additional file 1 **Table S1.** Optimization of hyperparameters of DeepM6ASeq. **Table S2.** Hyperparameter optimization for other classifiers. **Table S3.** Metrics of mean performance for tuning of hyperparameters. **Table S4.** Performance metrics for comparison of DeepM6ASeq with other classifiers on the mammalian unbalanced independent dataset. **Table S5.** Prediction scores at different confidence thresholds for species models. (PDF 100 kb)

Additional file 2 **Figure S1.** Performance of the mammalian model on the mammalian validation dataset. **Figure S2.** Cross-species performance. **Figure S3.** The clusters of learned motifs from RSAT for human, mouse and zebrafish. **Figure S4.** A comparison of percentages of potential bins in different categories for human, mouse and zebrafish. **Figure S5.** The distribution of nucleotide types of the most salienct nucleotides. **Figure S6.** The distribution of distances from the most salient non-A nucleotides to mapped miCLIP m6A. **Figure S7.** The distribution of the most salient A*s*. (PDF 469 kb)
