# Supplementary material for: DeepM6ASeq: prediction and characterization of m6A-containing sequences using deep learning
Source: BMC Bioinformatics. 2018 Dec 31;19(Suppl 19):524. doi: 10.1186/s12859-018-2516-4 (PMC6311933; doi:10.1186/s12859-018-2516-4)
Supplement: Supplementary file 1 — Table S1. Optimization of hyperparameters of DeepM6ASeq. Table S2. Hyperparameter optimization for other classifiers. Table S3. Metrics of mean performance for tuning of hyperparameters. Table S4. Performance metrics for comparison of DeepM6ASeq with other classifiers on the mammalian unbalanced independent dataset. Table S5. Prediction scores at different confidence thresholds for species models. (PDF 100 kb) [file 12859_2018_2516_MOESM1_ESM.pdf]

Table S1 Optimization of hyperparameters of DeepM6ASeq

Hyper-parameter	Choices
Maxpooling	0,2
Dropout ratio	0.25,0.5
Output units of BLSTM layer	$32 \times 2, 64 \times 2$
Neurons of FC layer*	32,64
Learning rate	0.01,0.001

*Number of neurons corresponds to 1/2 output units of BLSTM layer

Table S2 Hyperparameter optimization for other classifiers

Classifiers	Hyper-parameters optimization
RF	tree number: 300,400,500,600,700,800,900
LR	regularization strength: 0.001, 0.01, 0.1, 1, 10, 100, 1000; penalty: L1, L2
SVM	C: 1,10,100; gamma: 0.01,0.001; kernel: linear, polynomial, rbf

Table S3 Metrics of mean performance for tuning of hyperparameters

	Maxpooling	Dropout	Layers	Learning rate	AUROC	AUPR	Accuracy	MCC	F1-score
Model1	0	0.25	BLSTM:32; FC:32	0.001	0.846	0.833	0.768	0.538	0.775
Model2	0	0.25	BLSTM:32; FC:32	0.01	0.847	0.834	0.770	0.541	0.775
Model3	0	0.25	BLSTM:64; FC:64	0.001	0.847	0.835	0.771	0.543	0.777
Model4	0	0.25	BLSTM:64; FC:64	0.01	0.846	0.833	0.769	0.540	0.777
Model5	0	0.5	BLSTM:32; FC:32	0.001	0.848	0.835	0.772	0.544	0.776
Model6	0	0.5	BLSTM:32; FC:32	0.01	0.847	0.834	0.770	0.542	0.778
Model7	0	0.5	BLSTM:64; FC:64	0.001	**0.850**	**0.838**	0.772	**0.546**	0.777
Model8	0	0.5	BLSTM:64; FC:64	0.01	0.849	0.836	**0.773**	**0.546**	**0.780**
Model9	2	0.25	BLSTM:32; FC:32	0.001	0.844	0.830	0.767	0.536	0.776
Model10	2	0.25	BLSTM:32; FC:32	0.01	0.843	0.830	0.768	0.536	0.774
Model11	2	0.25	BLSTM:64; FC:64	0.001	0.844	0.832	0.767	0.535	0.775
Model12	2	0.25	BLSTM:64; FC:64	0.01	0.844	0.831	0.767	0.535	0.772
Model13	2	0.5	BLSTM:32; FC:32	0.001	0.848	0.836	0.771	0.543	0.777
Model14	2	0.5	BLSTM:32; FC:32	0.01	0.846	0.832	0.770	0.541	0.776
Model15	2	0.5	BLSTM:64; FC:64	0.001	0.849	0.836	0.771	0.542	0.776
Model16	2	0.5	BLSTM:64; FC:64	0.01	0.847	0.834	0.770	0.541	0.774

Table S4 Performance metrics for comparison of DeepM6ASeq with other classifiers on the mammalian unbalanced independent dataset

	Accuracy	F1-score	AUROC	AUPR	MCC
DeepM6ASeq	**0.763**	**0.687**	**0.841**	**0.725**	**0.505**
Random Forest	0.732	0.667	0.822	0.692	0.466
Logistic Regression	0.750	0.662	0.821	0.694	0.469
Support Vector Machine	0.740	0.654	0.815	0.687	0.453

The highest value for each accuracy measure is highlighted in bold.

Table S5 Prediction scores at different confidence thresholds for species models

	Moderate*	High*	Very high*
Mammalian	0.725	0.818	0.929
Human	0.772	0.841	0.920
Mouse	0.724	0.813	0.890
Zebrafish	0.715	0.820	0.90

*Moderate, High and Very high correspond to 90%, 95% and 99% specificity respectively.
