# Supplementary material for: DeepM6ASeq: prediction and characterization of m6A-containing sequences using deep learning
Source: BMC Bioinformatics. 2018 Dec 31;19(Suppl 19):524. doi: 10.1186/s12859-018-2516-4 (PMC6311933; doi:10.1186/s12859-018-2516-4)
Supplement: Supplementary file 2 — Figure S1. Performance of the mammalian model on the mammalian validation dataset. Figure S2. Cross-species performance. Figure S3. The clusters of learned motifs from RSAT for human, mouse and zebrafish. Figure S4. A comparison of percentages of potential bins in different categories for human, mouse and zebrafish. Figure S5. The distribution of nucleotide types of the most salienct nucleotides. Figure S6. The distribution of distances from the most salient non-A nucleotides to mapped miCLIP m6A. Figure S7. The distribution of the most salient As. (PDF 469 kb) [file 12859_2018_2516_MOESM2_ESM.pdf]

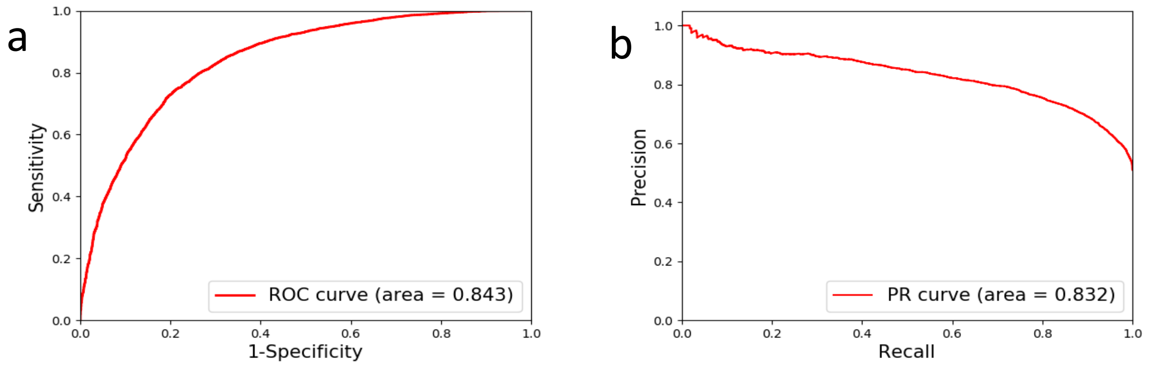

Figure S1 Performance of the mammalian model on the mammalian validation dataset. The performance is presented as a) a plot of ROC and b) a graph of a precision-recall.

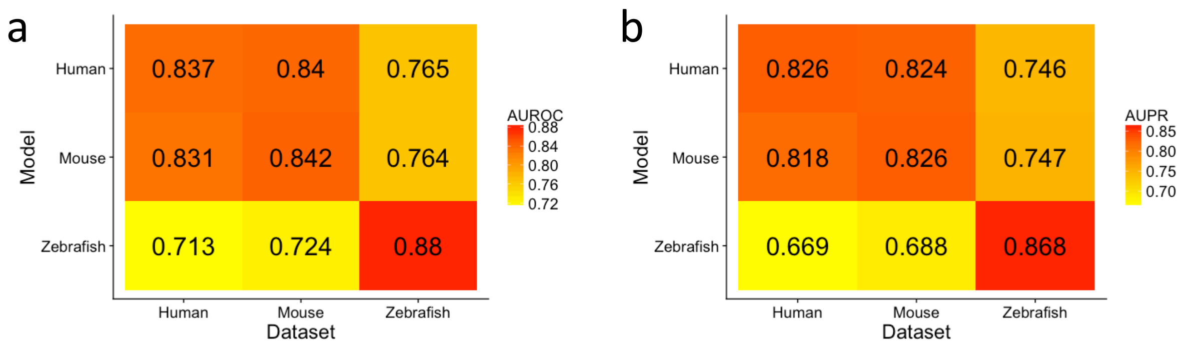

Figure S2 Cross-species performance. The rows of heatmaps represent a model type and the columns indicate a dataset type. Values in the heatmaps are a) AUROC b) AUPR.

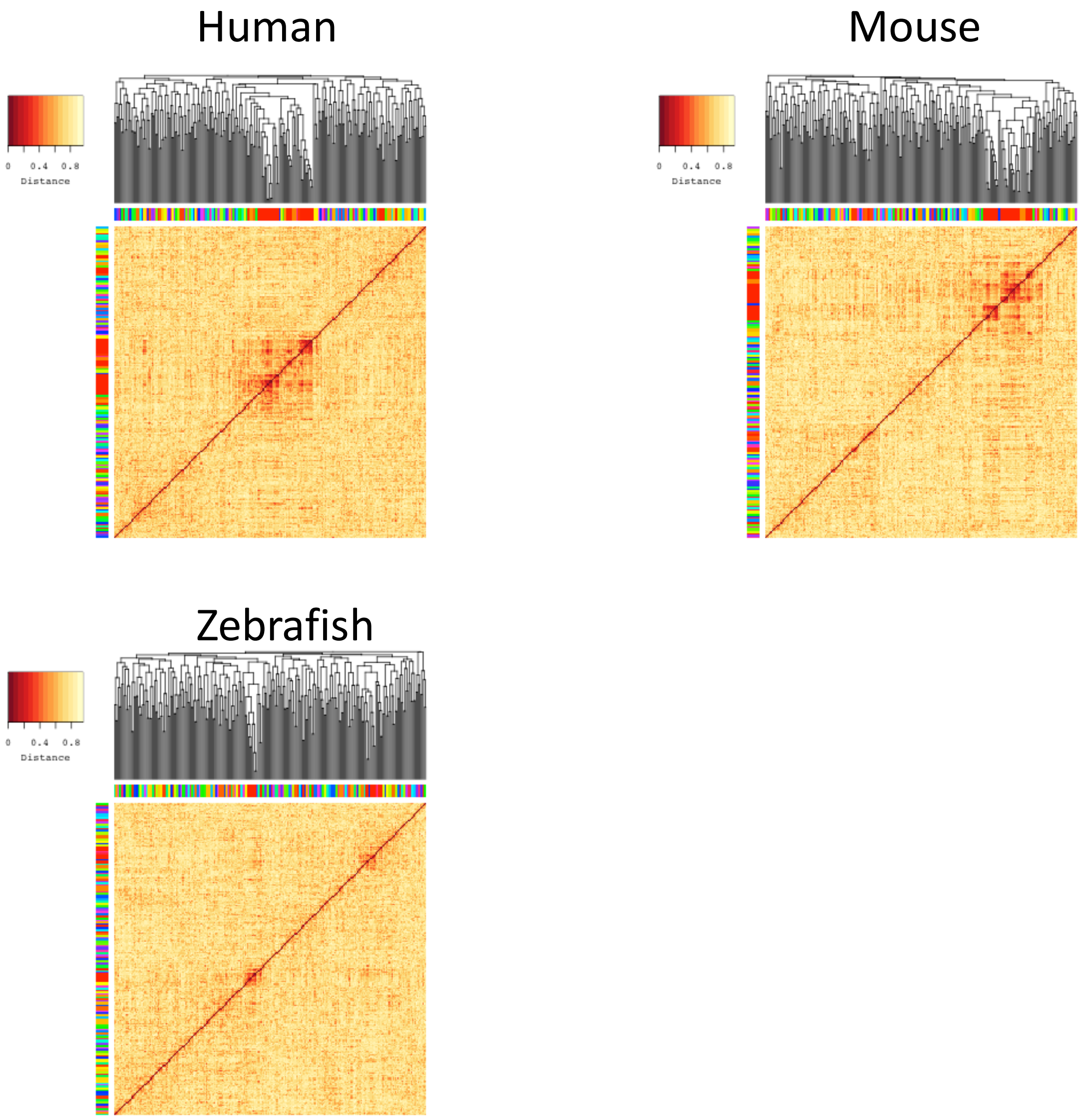

Figure S3 The clusters of learned motifs from RSAT for human, mouse and zebrafish. The blocks of different colors along the heatmaps represent different clusters.

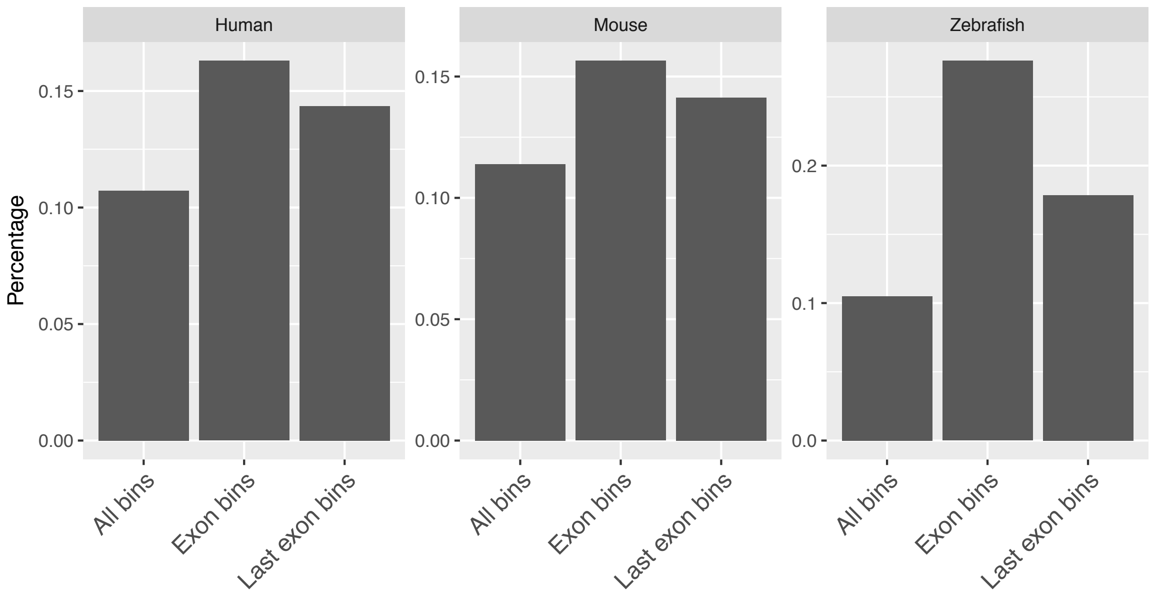

Figure S4 A comparison of percentages of potential bins in different categories for human, mouse and zebrafish. The X-axis represents different categories, including all bins, exon bins and last exon bins. Potential bins are the ones with confidence above the moderate threshold.

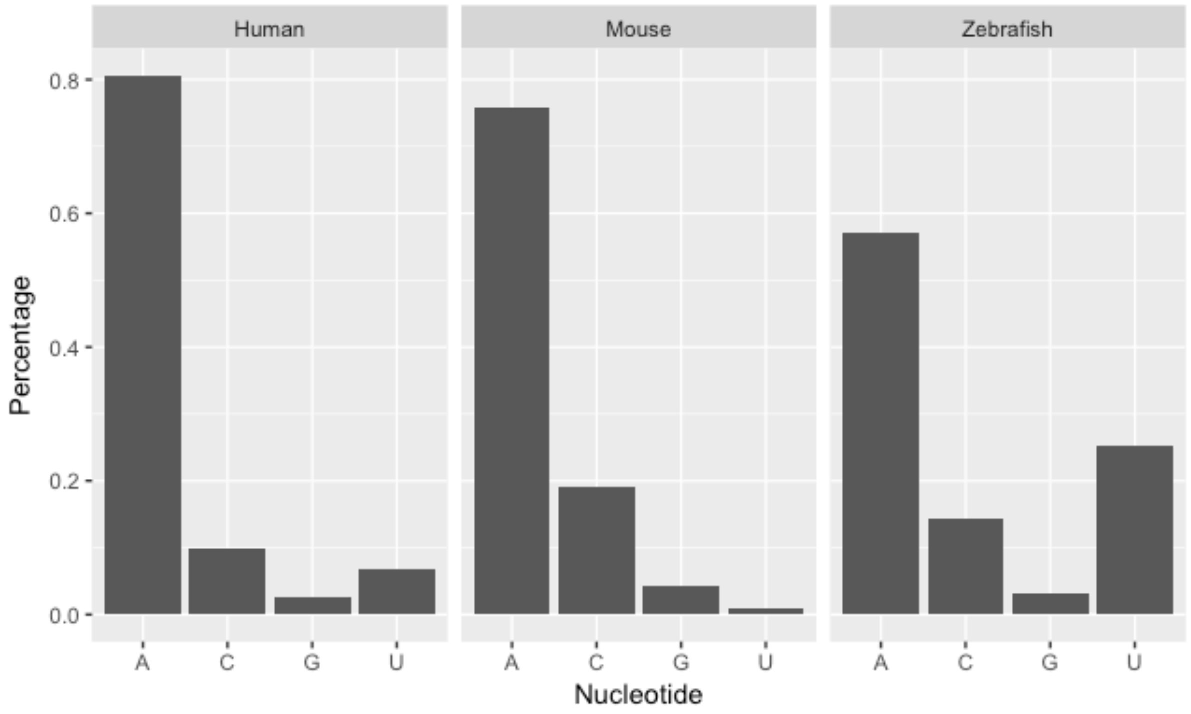

Figure S5 The distribution of nucleotide types of the most salienct nucleotides. The distribution of nucleotide types of the most salienct nucleotides in the independent test sequences with confidence above the moderate threshold for human, mouse and zebrafish. The X-axis represents the nucleotide types A, C, G and U.

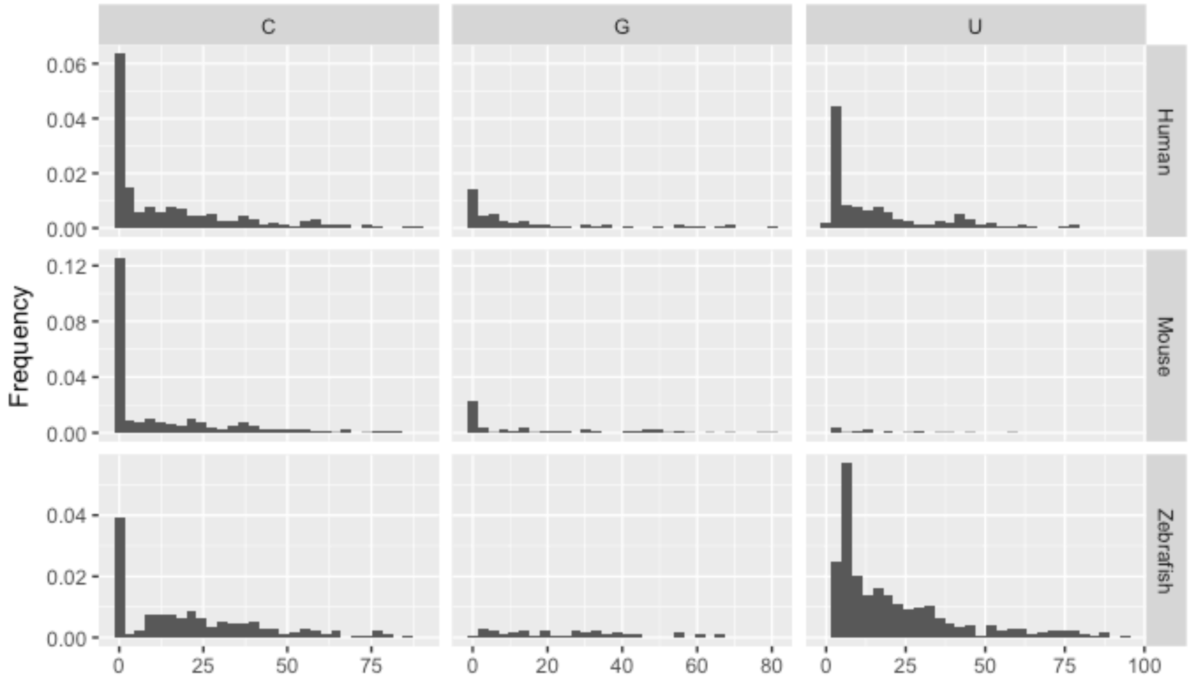

Figure S6 The distribution of distances from the most salient non-A nucleotides to mapped miCLIP m6A. The distribution of distances from the most salient non-A nucleotides in the independent test sequences with confidence above the moderate threshold to mapped miCLIP m6A in human, mouse and zebrafish. The X-axis denotes the distance.

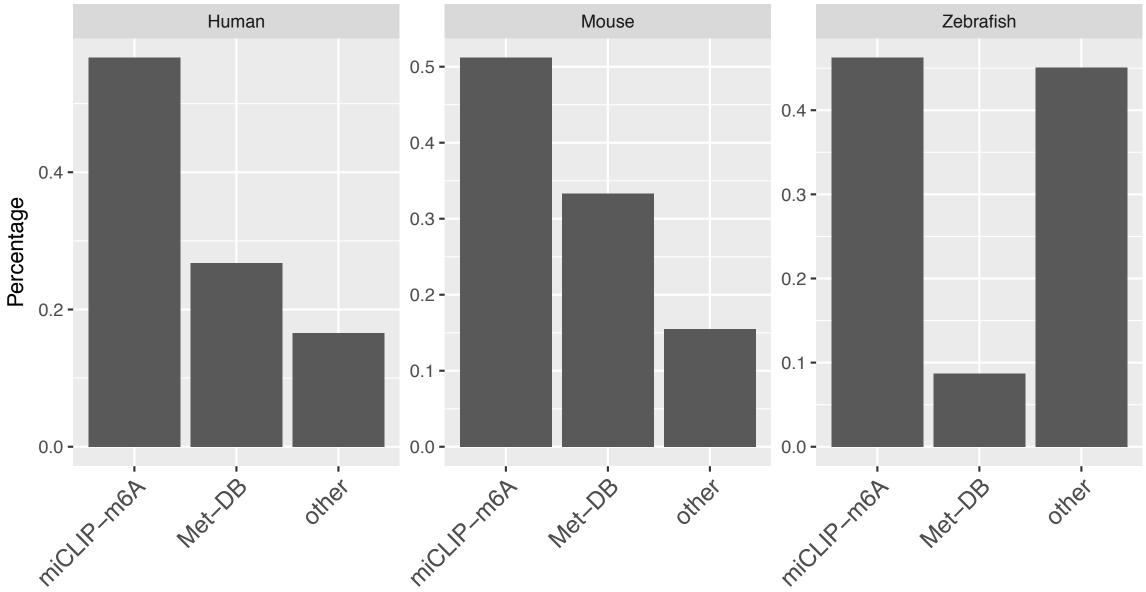

Figure S7 The distribution of the most salient A*s*. The distribution of the most salient A*s* in the independent test sequences with confidence above the moderate threshold by different

categories, including miCLIP-Seq m6A, Met-DB predicted m6A and the other in human, mouse and zebrafish.
